# Bacteriology, antibiotic treatment effect and adverse birth outcomes in pregnant women with and without bacteriuria: a registry study

**DOI:** 10.1007/s15010-025-02631-8

**Published:** 2025-09-10

**Authors:** Jon Dissing Sund, Mathilde Sif Frydensberg Nicolaisen, Jenny Dahl Knudsen, Michael Pedersen, Emil Hofman, Nina Weis, Ellen Moseholm

**Affiliations:** 1 Department of Infectious Diseases, Copenhagen University Hospital, Hvidovre, Hvidovre, Denmark; 2 Department of Clinical Microbiology, Copenhagen University Hospital, Rigshospitalet, Copenhagen, Denmark; 3 Department of Clinical Microbiology, Copenhagen University Hospital, Hvidovre, Hvidovre, Denmark; 4 Department of Clinical Medicine, Faculty of Health and Medical Sciences, University of Copenhagen, Copenhagen, Denmark

**Keywords:** Bacteriuria, Urinary tract infections, Pregnancy, Adverse birth outcome, Prenatal care, Urine culture

## Abstract

**Purpose:**

To investigate bacteriology, antibiotic treatment and adverse birth outcomes (ABOs) in pregnancies with and without bacteriuria and urinary tract infections (UTIs) based on urine cultures and clinical diagnoses.

**Methods:**

Registry-based cohort study. Population: Pregnancies with at least one urine culture analysed at one of two hospitals in the Capital Region, Denmark, between 2015 and 2021. Data were collected from clinical and national health registries. Descriptive statistics, t-tests, and logistic regressions were applied. Main outcome measures: Odds of ABOs (low birth weight (LBW), small for gestational age (SGA), prematurity), and the impact of antibiotic treatment.

**Results:**

74,253 pregnancies in 62,439 women with 178,599 urine cultures were included; 3,498 (4.7%) with a positive urine culture, of whom 2,786 had bacteriuria (no UTI/ASB diagnosis), 533 with a UTI diagnosis, and 179 with an ASB diagnosis, and 70,755 pregnancies without a significantly positive urine culture (comparison group). *Escherichia coli* (9.3%) was the most common uropathogen. Of included pregnancies, 43% received antibiotic treatments, and the average treatment timing was empirical. Bacteriuria and UTIs in pregnancy increased the odds of ABOs, and antibiotic treatment was associated with reduced odds. ASB was not associated with ABOs.

**Conclusion:**

Bacteriuria and UTIs in pregnancy, but not ASB, were significantly associated with ABOs and a lowering of odds of LBW when antibiotically treated. Our findings highlight the importance of pregnancy diagnostics, the consequences of bacteriuria, but also that further research on ASB is highly needed.

**Supplementary Information:**

The online version contains supplementary material available at 10.1007/s15010-025-02631-8.

## Introduction

To reduce complications for both mother and child, it is important to diagnose and treat infections during pregnancy efficiently. Urinary tract infections (UTIs), such as cystitis and pyelonephritis, are among the most common infections in pregnancy [1–3]. It is estimated that 1,000 pregnant women yearly are admitted to Danish hospitals due to UTIs [3], and UTIs during pregnancy have been shown to increase the risk of adverse birth outcomes (ABOs) [2, 4]. Significant bacteriuria is defined as ≥ 10^5 CFU (colony forming units) of the same bacterial strain pr. milliliter of urine, except for group B-Streptococci (GBS), which has lower CFU limits [2]. UTIs are defined as bacteriuria plus symptoms related to a UTI (fever, flank pain, dysuria, etc.). Asymptomatic bacteriuria (ASB) is defined as two urine cultures with significant bacteriuria, with no UTI-related symptoms in the anamnesis [1, 5, 6]. Therefore, significant bacteriuria may represent either a UTI or ASB, with ASB potentially preceding UTI.

During pregnancy, it is recommended to screen for and treat both asymptomatic and symptomatic bacteriuria with antibiotics to reduce the risk of adverse birth outcomes (ABOs) [3, 6–12]. However, strict criteria for sampling, treatment duration, follow-up, and diagnosis vary throughout guidelines [7, 12–14]. Danish recommendations state that positive or doubtful urine dipstick analysis should trigger a urine culture analysis for bacterial growth and antibiotic resistance patterns, and that a follow-up urine culture should be obtained upon any positive findings to confirm the bacterial strain [2].

The prevalences of bacteriuria and ASB in pregnant women in industrial countries are estimated at 5% and 2–10%, respectively [2, 3]. UTIs in pregnancy are relatively common, and cystitis (lower UTI) occurs in 1–2% of pregnancies [1, 2], whereas pyelonephritis (upper UTI) occurs in 0.3-2% of pregnancies [1–3, 6, 15]. *Escherichia coli* and *Streptococcus agalactiae* (GBS) are the most common uropathogens related to bacteriuria, UTIs, and ASB [5, 6, 15–19]. Approximations suggest that 20–30% of untreated ASB cases progress to pyelonephritis [3, 7, 20].

It is widely accepted that UTIs in pregnancy are associated with an increased risk of ABOs, including low birth weight (LBW) and prematurity [1, 3, 6, 13]. However, the evidence regarding the association between ASB and ABOs, as well as the effectiveness of antibiotic treatment in reducing the risk of ABOs in pregnant women with bacteriuria, is less certain [13]. A recent systematic review reported conflicting results regarding the association between ASB and LBW and preterm birth as well as the impact of treatment [21].

The study aimed to investigate bacteriology, the association between bacteriuria in pregnancy (i.e., bacteriuria without diagnosis, diagnosed UTI, and diagnosed ASB, respectively) and the odds of ABO, and the antibiotic treatment effect thereof by comparing women with bacteriuria to a matched comparison group without bacteriuria.

## Methods

### Study design and data sources

We conducted a registry-based cohort study utilizing urine culture data and data from Danish national health registries. Throughout the study, we utilized an encrypted 10-digit personal identification number (PIN) assigned to all Danish individuals at birth or upon approved immigration status [22] to extract and merge data from the following registries:

*The Department of Clinical Microbiology* (DCM) database contains information from urine cultures analysed at the Clinical Microbiology Departments at two University Hospitals (Hvidovre and Rigshospitalet) in the Capital Region of Denmark, including date, number, and results of urine culture analyses. The date of urine culture analyses and uropathogens were collected from this database.

*The National Patient Register* (NPR), established (est.) in 1976, contains data on somatic in- and out-patient hospitalizations [23]. Using the International Classification of Diseases 10th revision (ICD-10) classification [24]), we identified pregnancies with a UTI diagnosis (pyelonephritis, cystitis, and urethritis (other UTIs)), ASB diagnosis, and information on maternal comorbidities. Comorbidities included diabetes mellitus (type 1 and 2), gestational diabetes mellitus (GDM), chronic kidney disease (CKD), and hypertension. The comorbidities were included if the date of the ICD-10 coding in the NPR was within the period of 10 years prior to 8 weeks after birth. Selected ICD-10 codes for diagnosed UTIs, ASB, and comorbidities can be found in Supplementary Table S1.

*The Register of Pharmaceutical Sales* (est. 1994) and *The National Hospital Medication Register* (est. 2018) [25] contain data on dispensed medication from Danish pharmacies and prescriped hospital medication, respectively. Using the Anatomical Therapeutic Chemical classification (ATC) code, we collected information on antibiotic treatments in pregnancy (ATC code J01).

*The Medical Birth Register* (est. 1973) contains data on pregnancy, birth, and the newborn child [26]. Date of birth, gestational age (GA), birthweight, antenatal smoking, and maternal age, weight, and height were collected.

### Study population

**Fig. 1:**
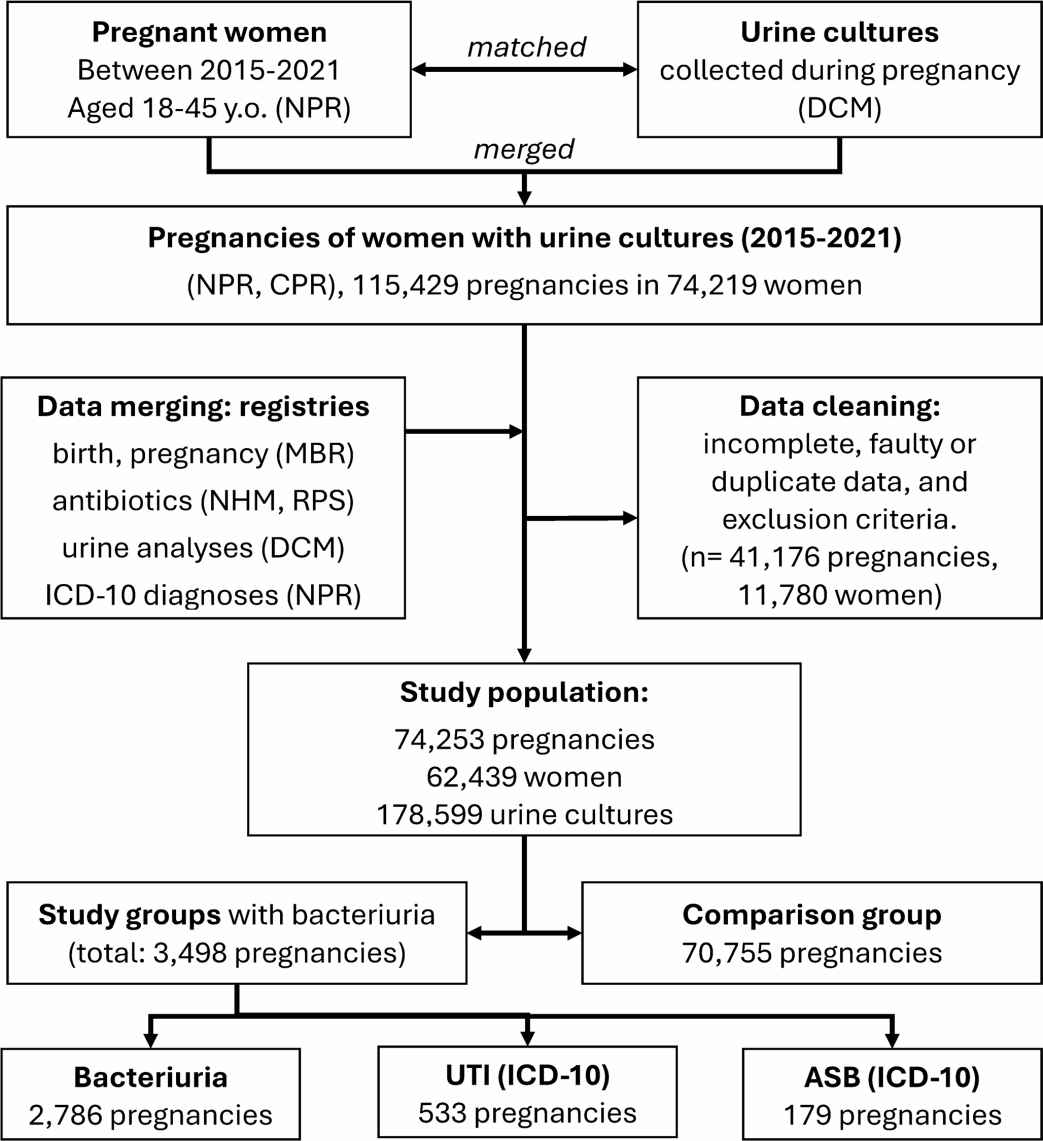
Study population flowchart. The study population flowchart was created in Microsoft PowerPoint. NPR (The National Patient Register), CPR (The Danish Civil Registration System), MBR (The Medical Birth Register), NHM (The National Hospital Medication Register), RPS (The Register of Pharmaceutical Sales)

Inclusion criteria for the study population were: pregnant women, 18–49 years of age, living in the catchment area of Copenhagen University Hospitals, Hvidovre and Rigshospitalet, Copenhagen, Denmark, with singleton births, and at least one urine culture analysis during pregnancy between 2015 and 2021 registered in the DCM database. Exclusion criteria were: pregnancies with incomplete or missing data, birth GA < 22 weeks, stillbirths, and non-singleton births. Cases with a registered diagnosis of ASB or a UTI, but without a time-relevant, a negative or an inconclusive sample, were excluded. Figure 1 shows the study population presented in a flow chart.

### Study groups

The study population was divided into four mutually exclusive groups.

- A *bacteriuria* group including pregnancies in women with at least one significantly positive urine culture (> 10^5 CFU/mL urine) during pregnancy in the DCM database, but without an ICD-10 diagnosis for a UTI or ASB registered in the NPR during pregnancy.
- A *UTI group* including pregnancies in women registered with at least one significantly positive urine culture (> 10^5 CFU/mL urine) during pregnancy in the DCM database, and with an ICD-10 diagnosis of UTI registered in the NPR (pyelonephritis, cystitis, and urethritis (other UTIs)) during pregnancy.
- An *ASB group* including pregnancies in women with at least one significantly positive urine sample during pregnancy, registered in the DCM database, and an ICD-10 diagnosis of ASB (DN390A and DN390) registered in the NPR during pregnancy.
- A *comparison group* including pregnancies in women who had at least one urine culture analysis during pregnancy, but with insignificant or no bacterial growth and without a registered ICD-10 diagnosis of ASB or UTI in the NPR during pregnancy.

Cases with a registered ASB diagnosis that were later diagnosed with a UTI were assigned to the exposure group (ASB or UTI) that had a corresponding urine culture result, to reduce the risk of misclassification. Sensitivity analyses were performed to evaluate the impact of including these cases.

Pregnancies that had cultures matching both diagnoses were excluded. All groups and analyses were checked for duplicate case representation. Women with an ICD-10 code for UTI/ASB and a negative, inconclusive or no corresponding urine sample (*n* = 1,639) were excluded to enhance diagnostic specificity between ASB and UTIs, and distinct groups.

### Outcome

The main outcome was ABOs defined from the guidelines of the Capital Region of Denmark [14]. Low birth weight (LBW) was defined as birth weight being < 2,500 g. Small for gestational age (SGA) was defined as a birth weight more than 2 standard deviations below the average weight of children born at that GA in the Danish population [27]. Prematurity was defined as birth before GA 37 + 0 weeks. Furthermore, we defined a composite outcome “any adverse outcome” defined as the occurrence of any of the three selected ABOs.

Antibiotic treatment was defined as a registered antibiotic treatment being either prescribed at a hospital or dispensed at a pharmacy between the sampling date and 5 days after urine culture results became available.

### Statistical analyses

Descriptive statistics were applied in describing the study population with categorical variables as counts (%) and continuous variables as means with standard deviations (SD). Differences in baseline characteristics were analysed through chi-squared tests and Fischers Exact test. Proportion calculations were used for antibiotic use during pregnancy.

Adjusted and unadjusted logistic regression models were applied to examine the association between ABOs and bacteriuria, UTI, and ASB, respectively, in addition to the treatment effect. The results are reported as odds ratios (OR; either as crude OR (cOR) or adjusted OR (aOR)). Multivariate models included adjustment for covariates of maternal age, body mass index (BMI), parity, comorbidities, maternal smoking, antibiotics during pregnancy, and geographical region of origin entered into the model in one single step. A 95% confidence interval ([95% CI]) was employed, with *P*-values of 0.05 as the threshold for statistical significance. Analysis was conducted using the open source software “R”, version 4.3.3, and employed packages dplyr, broom and epitools.

### Ethics

This study was approved by the Danish Data Protection Agency (P-2022-862). All analyses were conducted on pseudonymized datasets with no access to identifiable information. All data were stored in accordance with the Danish Data Protection Council Guidelines. Ethical approval was not required as no new analyses were conducted on the urine samples.

## Results

### Study population characteristics

The study population consisted of 74,253 pregnancies in 62,439 women, with a total of 178,599 urine cultures obtained during pregnancy identified within the study period. The final study population included 3,498 pregnancies (4.7%) with significant bacteriuria and 70,755 pregnancies without bacteriuria in pregnancy (comparison group). Of the 3,498 pregnancies with significant bacteriuria, 2,786 (4.7%) had significant bacteriuria alone without an ICD-10 diagnosis of UTI/ASB, 533 (0.72%) had an ICD-10 diagnosis of a UTI, while 179 (0.24%) had an ICD-10 diagnosis of ASB.

The characteristics of the study population are presented in Table 1. No statistically significant differences were observed between the UTI and ASB groups. The UTI and ASB groups were significantly younger than the bacteriuria and comparison groups. The UTI group more often had diabetes (type 1 and 2), GDM, hypertension, or CKD, and a higher prevalence of smoking than the comparison group. The UTI group more often had diabetes and hypertension than the bacteriuria group. No statistically significant differences were found between the bacteriuria and comparison group, except that women in the bacteriuria group smoked more.

**Table 1:** Demographic characteristics 74,074 pregnancies in 62,270 women having at least one urine culture taken during pregnancy between 2015–2021 urinary tract infection (UTI), bacteriuria, and a comparison group of 70,755 pregnancies without a UTI or bacteriuria

Study groups: ^1^	UTI			Bacteriuria		Comparison group	
	*n* = 533			*n* = 2,786		*n* = 70,755	
**Age**: n (%), mean [SD] ^2^	30 [5.9]			31.1 [5.14]		31.4 [4.6]	
< 20	13 (2.4)			22 (0.78)		191 (0.3)	
20–24	83 (15.6)			260 (9.3)		3,939 (5.6)	
25–29	170 (32)			807 (28.9)		20,811 (29.4)	
30–34	139 (26.1)			1,000 (35.9)		28,263 (39.9)	
35–39	89 (16.7)			525 (18.8)		14,220 (20.1)	
> 40	39 (7.3)			172 (6.17)		3,331 (4.7)	
**Parity**: n (%), mean [SD] ^2^	1.6 [0.9]			1.56 [0.85]		1.6 [0.8]	
Uniparous	217 (40.7)			1,084 (38.9)		39,151 (55.3)	
Multiparous	316 (59.3)			1,702 (61.1)		31,594 (44.6)	
No available data	0 (0)			0		10 (0.01)	
**BMI**: n (%), mean [SD] ^2^	24.3 [11.2]			23.8 [6]		23.7 [4.5]	
Underweight (< 18.5)	38 (7.1)			156 (5.6)		3,031 (4.3)	
Normal weight (18.5–24.9)	305 (57.2)			1,776 (63.7)		45,940 (64.9)	
Overweight (25-29.9)	119 (22.3)			545 (19.6)		14,344 (20.3)	
Obesity (> 29.9)	56 (10.5)			272 (9.6)		6,537 (9.2)	
No available data	15 (2.8)			37 (1.3)		903 (1.3)	
**Diabetes mellitus**: n (%) ^3^							
Yes	13 (2.4)			25 (0.9)		496 (0.7)	
**GDM**: n (%) ^3^							
Yes	32 (6)			138 (4.9)		2,771 (3.9)	
**Chronic renal disease**: n (%) ^3^							
Yes	NA			6 (0.2)		61 (0.1)	
**Hypertensive disorder**: n (%) ^3^							
Yes	32 (6)			136 (4.9)		3,075 (4.3)	
**Smoking during pregnancy**: n (%) ^4^							
Yes	74 (13.8)			282 (10.1)		4,741 (6.7)	
Unknown	0 (0)			0		3243 (4.6)	
**Antibiotics during pregnancy**: n (%) ^4^							
Yes	506 (94.9)			2,490 (89,4)		31,273 (44.2)	
**Region of origin**: n (%) ^4^							
Europe	381 (71.5)			2,137 (76.7)		58,811 (83.1)	
The Middle East	70 (13.3)			285 (10.2)		4,770 (6.7)	
Asia + Oceania	45 (8.4)			196 (7)		4,482 (6.3)	
Africa	25 (4.7)			120 (4.3)		1,813 (2.6)	
Americas	12 (2.3)			48 (1.7)		879 (1.3)	

^**1**^**UTI group**: women with at least one significantly positive urine culture during pregnancy and an ICD-10 code for a UTI. **Bacteriuria group**: women with at least one significantly positive urine culture during pregnancy and no ICD-10 code for ASB (asymptomatic bacteriuria)/UTI. **Comparison group**: women with no bacteriuria, UTI and ASB. ^**2**^Age: grouped (outliers < 15 and > 49 excluded). BMI (Body mass index): values < 15 and > 65 added to ”no available data” as outliers or typos. Parity: grouped registered parity. ^3^Hypertension, gestational diabetes mellitus (GDM), diabetes mellitus, and chronic renal disease (CKD): ICD-10 diagnosed within 10 years before and 8 weeks after birth. ^4^Smoking: any history of smoking during pregnancy. Antibiotics during pregnancy: ATC-coded drug prescription or administration during pregnancy. Region of origin: ISO-3166 codes grouped regionally. Note: “NA” cells (Not Applicable) refer to cells with low observations to secure anonymity. Note: The table was created using the open-source program R

### Distribution of uropathogens in urine cultures and antibiotic treatment patterns

Table 2 shows that *E. coli* was the most common uropathogen isolated in women with bacteriuria, UTI, and ASB. However, mixed flora, defined as three or more bacterial agents identified, was the most frequent urine culture result in the bacteriuria (22.6%) and ASB groups (32.4%).

**Table 2:** Distribution of bacterial uropathogen growth in urine samples of the study population and treatment patterns in pregnancies involving antibiotics. UTIs (*n* = 533 pregnancies, 506 treated, 1,310 treatments), bacteriuria (*n* = 2,786 pregnancies, 2,482 treated, 5,310 treatments), and the comparison group (*n* = 70,755 pregnancies, 30,753 treated, 45,181 treatments)

Bacterial uropathogen growth	Study population group ^1^		
Number of pregnancies	UTI *n* = 533	Bacteriuria *n* = 2,786	Comparison group *n* = 70,755
	(%)	(%)	(%)
Mixed flora ^2^	21.8	22.6	31.7
*Streptococcus agalactiae*, (GBS)	5.3	8.7	3.8
*Escherichia coli*	28.9	22	3.6
Mixed flora (skin/mucus)	1.2	1.8	1.7
*Enterococcus spp*.	1.9	1.4	1.1
*Enterococcus faecalis*	4.8	3.6	0.8
Others	13.3	11.3	2.9
Total positive %	77.2	71.4	45.6
Number of samples	2,662	9,724	165,509

**Treatment patterns**	Study population group ^1^		
Antibiotics prescribed ^3^	UTI n (%)	Bacteriuria n (%)	Comparison group n (%)
Pre-culture:	334 (25)	1,194 (22)	8,268 (18)
On-date:	194 (15)	654 (12)	2,666 (6)
Post-culture:	301 (23)	1,175 (22)	5,286 (12)
Other	481 (37)	2,287 (43)	28,960 (64)
No. of treatments	1,310	5,310	45,181
Mean time difference ^4^	-2.63 days	-2.69 days	-2 days
Treatments pr. Pregnancy	2.6	2.1	1.47
% of population treated	95%	89%	43%

^**1**^ **UTI group**: women with at least one significantly positive urine culture during pregnancy and an ICD-10 code for a UTI. **Bacteriuria group**: women with at least one significantly positive urine culture during pregnancy and no ICD-10 code for ASB/UTI. **Comparison group**: women without an ASB diagnosis, a UTI diagnosis or bacteriuria. ^**2**^Urine culture analyses with three or more undistinguished bacterial strains. ^3^Pre-culture”: Antibiotics prescribed between sampling date and culture growth results. “On-date”: Antibiotics prescribed the same date as available culture growth results. ”Post-culture”: Antibiotics prescribed between culture growth results and five days later. ”Other”: treatment lies outside given periods. ^4^Time difference in days between urine culture results and prescribed antibiotics. A negative value indicates that antibiotics were on average prescribed before urine culture results were available. Note: this table is not limited to significantly positive urine samples for the comparison group, but instead any amount of CFU/mL. Note: The analysis was performed using the open-source program R

On average, all study groups received antibiotic treatment prior to urine culture results being available, indicating that the treatment was empirical (Bacteriuria: 2.7 days before, UTI 2.6 days before, and ASB 2 days before). (Table 2).

All study groups were on average treated more than once pr. pregnancy (bacteriuria (2.1 treatments), UTI (2.6 treatments), ASB (1.9 treatments), and the comparison group (1.5 treatments)). (Table 2).

43% of pregnancies in the comparison group involved antibiotics at some point during pregnancy. In accordance with guidelines, 89%, 95%, and 94% of the bacteriuria, UTI, and ASB groups, respectively, were treated [20] (Table 2).

### The odds of adverse birth outcomes in pregnancies with bacteriuria, utis, and ASB

Table 3 shows the ORs of ABOs in pregnancies with bacteriuria or UTI in an adjusted logistic regression analysis compared to the comparison group. Bacteriuria was significantly associated with LBW (aOR 1.4 [95% CI, 1.2–1.7]), prematurity (aOR 1.3 [1.1–1.5]), and any adverse outcome (aOR 1.2 [95% CI, 1.1–1.4]), but not SGA. UTIs were significantly associated with LBW (aOR 2.6 [95% CI, 1.9–3.5]), prematurity (aOR 2.3 [1.7-3]), and any adverse outcome (aOR 1.6 [95% CI, 1.3–1.9]), but not SGA. Of the ASB group, 21% were subsequently ICD-10 diagnosed with a UTI without any time-relevant urine cultures (65% cystitis, 22% pyelonephritis) (Supplementary Table S2). Sensitivity analyses excluding these cases did not change the results significantly. The most common ICD-10 diagnosis in the UTI group was cystitis (69% of diagnoses).

**Table 3:** Adjusted logistic regression analysis on adverse birth outcomes (LBW, SGA, and prematurity) among pregnancies with bacteriuria or a urinary tract infection (UTI) compared to women without a significantly positive urine culture (the comparison group; *n* = 70,755)

Variable	Pregnancies with bacteriuria (*n* = 2,786)									Pregnancies with UTI (urine cultures + ICD-10 diagnosis) (*n* = 533)																						
	LBW^1^		SGA^1^		Prematurity			Any outcome^1^					LBW					SGA					Prematurity					Any outcome				
	aOR^2^ (95% CI)	*p*	aOR (95% CI)	*p*		aOR (95% CI)	*p*		aOR (95% CI)		*p*		aOR (95% CI)		*p*		aOR (95% CI)			*p*		aOR (95% CI)			*p*		aOR (95% CI)			*p*		
Group	1.4 (1.2–1.7)	< 0.01	1.1 (0.99–1.3)	0.06		1.3 (1.1–1.5)	< 0.01		1.2 (1.1–1.4)		< 0.01	2.6 (1.9–3.5)		< 0.01		1.3 (0.95–1.6)			0.1		2.3 (1.7-3)			< 0.01		1.6 (1.3–1.9)			< 0.01			
**Maternal data**																																
Age	1.02 (1.01–1.03)	< 0.01	1.02 (1.01–1.02)	< 0.01		1.01 (1.01–1.02)	< 0.01		1.01 (1-1.02)		< 0.01	1.02 (1.01–1.03)		< 0.01		1.1 (1.01–1.2)			< 0.01		1.01 (1-1.02)			< 0.01		1.01 (1-1.02)			< 0.01			
BMI	1 (0.99–1.01)	0.2	0.97 (0.97–0.98)	< 0.01		1 (0.99–1.01)	0.1		0.99 (0.98–0.99)		< 0.01	1 (0.99–1.01)		0.3		0.98 (0.97–0.98)			< 0.01		1 (0.99–1.01			0.2		0.99 (0.98–0.99)			< 0.01			
Parity	0.8 (0.7–0.8)	< 0.01	0.7 (0.65–0.71)	< 0.01		0.84 (0.8–0.9)	< 0.01		0.74 (0.7–0.8)		< 0.01	0.76 (0.7–0.8)		< 0.01		0.68 (0.65–0.7)			< 0.01		0.83 (0.8–8.8)			< 0.01		0.73 (0.71–0.76)			< 0.01			
Smoking	1.7 (1.5–1.95)	< 0.01	1.5 (1.4–1.7)	< 0.01		1.5 (1.3–1.6)	< 0.01		1.5 (1.4–1.7)		< 0.01	1.7 (1.5-3)		< 0.01		1.5 (1.4–1.7)			< 0.01		1.5 (1.4–1.7)			< 0.01		1.5 (1.4–1.7)			< 0.01			
**Comorbidities**																																
Diabetes	2.2 (1.6-3)	< 0.01	0.7 (0.5–1.1)	0.1		4.8 (3.8–5.9)	< 0.01		2.4 (1.9–2.9)		< 0.01	2.2 (1.6-3)		< 0.01		0.8 (0.5–1.1)			0.13		4.4 (3.5–5.5)			< 0.01		2.2 (1.8–2.7)			< 0.01			
Hypertension	2.2 (1.9–2.5)	< 0.01	1.4 (1.4–1.8)	< 0.01		1.6 (1.4–1.8)	< 0.01		1.5 (1.3–1.6)		< 0.01	2.1 (1.8–2.4)		< 0.01		1.6 (1.4–1.7)			< 0.01		1.6 (1.4–1.8)			< 0.01		1.5 (1.3–1.6)			< 0.01			
CKD	4.5 (2.4–8.4)	< 0.01	1.2 (0.6–2.5)	0.7		5.4 (3–10)	< 0.01		2.8 (1.6–6.7)		< 0.01	5 (2.5–9.4)		< 0.01		1.1 (0.5–2.5)			0.8		5.7 (3.2–10.4)			< 0.01		2.6 (1.5–4.6)			< 0.01			
GDM	1.03 (0.9–1.3)	0.7	1 (0.9–1.3)	0.8		1.3 (1.1–1.5)	< 0.01		1.1 (0.98–1.2)		0.1	0.97 (0.9–1.2)		0.78		0.97 (0.9–1.1)			0.7		1.2 (1.1–1.4)			< 0.01		1.1 (0.96–1.2)			0.2			

^**1**^LBW = Low birth weight, SGA = Small for gestational age, Any outcome = the occurrence of any of the three adverse birth outcomes (LBW, SGA and prematurity). ^**2**^aOR (adjusted odds ratio) adjusted for: age, BMI, parity, diabetes mellitus, hypertension, chronic kidney disease (CKD), gestational diabetes mellitus (GDM), and smoking. Note: The statistical analysis was performed using the open-source program R

Increasing parity was associated with a decrease in odds of ABOs. Comorbidities as diabetes, GDM, CKD, and hypertension mainly showed to increase the odds of ABOs. (Unadjusted results are presented in in Supplementary Tables S3.1 and S3.2). Odds of ABOs in UTI pregnancies with diagnoses cystitis, pyelonephritis and other ICD-10 diagnoses are presented in Supplementary Table S4. The crude number and prevalence of adverse birth outcomes in the study population groups are presented in Supplementary Table S5.

Table 4 shows the effect of antibiotic treatment on the odds of ABOs. A reduction in odds of ABOs was seen when treating both bacteriuria (SGA, prematurity and any adverse birth outcome) and UTIs (LBW).

**Table 4:** Adjusted logistic regression showing the effect of antibiotic treatment of bacteriuria and utis on the odds of adverse birth outcomes (LBW, SGA, and prematurity) among pregnancies with bacteriuria (*n* = 2,786, 2,482 treated) or a urinary tract infection (UTI, *n* = 533, 506 treated), comparing treated pregnancies with untreated pregnancies

		LBW ^1^		SGA ^1^		Prematurity		Any adverse birth outcome ^1^	
**Group**	**n**	aOR ^2^ (95% CI)	*P*	aOR (95% CI)	*P*	aOR (95% CI)	*P*	aOR (95% CI)	*P*
Bacteriuria ^3^	2,786	0.7 (0.5–1.2)	0.2	0.7 (0.5–0.95)	0.03	0.6 (0.4–0.9)	0.01	0.6 (0.5–0.8)	< 0.01
UTI ^3^	533	0.3 (0.1–0.8)	0.02	0.53 (0.2–1.5)	0.23	0.4 (0.15–1.1)	0.07	0.2 (0.05–1.04)	0.06

^**1**^LBW = Low birth weight, SGA = Small for gestational age, Any outcome = the occurrence of any of the three adverse birth outcomes (LBW, SGA and prematurity). ^**2**^aOR (adjusted odds ratio) adjusted for: age, BMI, parity, diabetes mellitus, hypertension, chronic kidney disease (CKD), gestational diabetes mellitus (GDM), and smoking. ^**3**^Bacteriuria (significantly positive urine culture and no ICD-10 diagnosis of UTI or ASB). UTI (urinary tract infection, significantly positive urine culture and an ICD-10 code for a UTI). Note: The statistical analysis was performed using the open-source program R

There was no significant associations between ASB and any of the assessed ABOs, and no significant results in terms of antibiotic treatment effect for ASB (Supplementary table S6 and S7).

## Discussion

### Main findings

In this registry study on ABOs in women with bacteriuria during pregnancy, we found that both bacteriuria and UTIs were associated with increased odds of ABOs. Antibiotic treatment reduced the odds of ABOs for bacteriuria and UTIs. *E. coli* was the most common uropathogen, while mixed flora was the most frequent urine culture finding. A substantial proportion of antibiotic treatments were empiric rather than targeted.

The prevalence of bacteriuria in the total population of 4.7% was slightly lower than found in a previous Danish study [12].

E.coli being the most common uropathogen is consistent with previous literature [5, 6, 15–19]. Mixed flora samples introduce uncertainty, as they often result from contamination due to improper sampling. However, it cannot be entirely excluded that such samples may mask the presence of a treatable uropathogen or indicate a UTI requiring further investigation.

Our finding that nearly half of all pregnancies in the comparison group involved antibiotic treatment is similar to that of previous literature [28], and raises concerns on potential over-treatment and potential development of antibiotic resistance, especially in light of the current lack of evidence on the treatment effect of bacteriuria, ASB, and UTIs [13, 29]. The high proportion of antibiotic use may also be influenced by factors such as the presence of other infectious diseases, empirical treatments, or treatments initiated based solely on a positive dipstick result. This highlights the need for further research to understand the drivers behind antibiotic use in this population and to develop guidelines that minimize unnecessary treatments.

Since previous literature and guidelines generally call for targeted treatment [1, 2, 6, 13], our findings on the frequency of empirical treatment highlight a discrepancy between guidelines and clinical practice. Likewise, potential side effects of antibiotics, like fungal infections and gastrointestinal issues, appear to be rarely addressed in the literature [13].

Our finding that both bacteriuria and UTIs were associated with increased odds of ABOs supports those from previous studies [5, 8], and in particular, current UTI treatment- and screening guidelines [2, 14].

Discrepancies in the effect of treating bacteriuria on the risk of ABOs have, however, been reported in the literature [13, 21, 29]. While treating bacteriuria is not unequivocally beneficial, treating symptomatic UTIs appears more clearly favorable. Hypothetically, treating bacteriuria before it progresses to a UTI may reduce hospitalizations and ABOs caused by the infection. In our study, antibiotic treatment during pregnancy in women with bacteriuria was associated with lower odds of SGA and prematurity, but not LWB, supporting the potential benefit of treatment. However, a recent meta-analysis reported an association between significant bacteriuria in pregnancy and preterm delivery and LBW, but not SGA, and reported that antibiotics did not significantly alter these outcomes [29].

The incongruence between our findings and current literature regarding the effect of treatment on SGA and prematurity in UTI cases may be explained by the small sample size of the untreated population in our study [3, 13]. Associations between confounding variables, such as smoking, increasing age, and increasing BMI, and comorbidities including diabetes, gestational diabetes mellitus, chronic kidney disease, and hypertension with ABOs, were consistent with previous literature [1, 16], highlighting the importance of attention regarding these factors. Surprisingly, increasing parity showed a protective effect against ABOs. This protective effect was more pronounced in our study than previously reported [1, 16].

For ASB, we did not find an association with odds of ABOs and the effect of treatment [1, 2, 21, 30]. While associations between ASB and pyelonephritis, and pyelonephritis and ABOs, respectively, have been reported in the literature, associations between ASB and ABOs are not clear [6, 7, 16, 21].

The natural history of bacteriuria in pregnancy, as well as the progression from ASB to UTI, has not been thoroughly studied. Moreover, there is no conclusive evidence of a definitive association between bacteriuria and subsequent ABOs. Overall, our understanding of these disease progressions and the effects of antibiotic treatment at different stages of bacteriuria is limited. As one review argued [16], the treatment effect of bacteriuria and ASB cannot be reliably assessed through randomization until the causal relationships between bacteriuria, ASB, and UTIs are better understood. This presents an ethical dilemma: randomizing participants to a placebo group may pose potential risks for ABOs, even if those risks are not yet conclusively established.

### Strengths and limitations

Our study has strengths in its large study population and the high data quality provided by Danish health registries. These registries are known for their comprehensive and prospectively collected data, which enhances the reliability and validity of our findings [22, 23, 25, 26]. This study has a strong clinical relevance by addressing common infections in pregnancy that affect thousands of women yearly. It includes detailed clinical data and diagnostics, yielding results that applies to obstetric and prenatal care.

This study also has some limitations. While over 40,000 pregnancies were excluded, they were so due to exclusion criteria, and missing or imcomplere key birth and maternal data, and were so to enhance data quality. The study population remains large and demographically diverse, including two hospitals with a variety of patient groups. Nevertheless, findings may not be fully generalizeable to national settings.

Some urine samples, treatments, and pregnancies were excluded due to faulty or missing data, highlighting the reliance on accurate diagnostics and registry data. In general, registry data provides only a snapshot view of the clinical situation, and lacks information on underlying decision-making and disease progression. Additionally, the inability to distinguish specific uropathogens within the “mixed flora” cultures presents uncertainty, especially regarding whether cases were repeated, treated, or resulted in a diagnosis.

Our analysis included only ICD-10 diagnoses registered in the hospital sector, which likely missed ASB cases diagnosed and managed in general practice or prenatal care. This limitation may lead to an underestimation of ASB prevalence and affect the generalizability of our findings, as hospital-registered cases might represent more severe or complicated infections compared to those treated outside hospitals. The low ASB prevalence in the registries likely reflects incomplete ICD-10 registration or challenges in transferring data between healthcare sectors, such as ASB and UTI diagnoses that should be visible across sectors. This indicates that these data alone are insufficient for studying ASB. Consequently, we plan a prospective cohort study including the primary care sector to collect comprehensive ASB data for more robust analyses.

In line with this, a potential limitation of the study is the risk of misclassification bias between ASB cases that precede UTI cases, with one or both diagnoses potentially being recorded. Although we addresses this statistically through sensitivity analyses to maintain distinct groups, misclassification cannot be completely ruled out. Misclassification due to symptom presentation, clinical practice, and decision-making is still possible.

## Conclusion

In this registry study, the prevalence of bacteriuria was 4.7%, and the most common uropathogen was E. coli, with mixed flora being the most frequent urine culture result. Pregnant women were often treated with antibiotics, with a majority of treatments being empirical. Bacteriuria and UTIs were associated with ABOs, and antibiotic treatment was associated with reduced odds of these outcomes. ASB was not associated with ABOs.

These findings highlight a knowledge gap regarding ASB and its relationship with ABOs. They also suggest that registry data alone may be insufficient to provide definitive evidence in this area.

We encourage accurate and systematic urine sampling along with a thorough symptom assessment during pregnancy, especially by the general practitioner. Furthermore, we propose conducting prospective studies on ASB, a potential precursor to one of the most common and problematic infections during pregnancy, UTIs, and on the associations between bacteriuria, ASB, and ABOs, to better understand their interplay and ultimately to improve prenatal care.

## Supplementary Information

Below is the link to the electronic supplementary material.

Supplementary Material 1

## Data Availability

Data used in this study was drawn from Danish national health care registries. According to Danish law, these data in their raw form cannot be shared publicly.
