# Supplementary material for: Bacteriology, antibiotic treatment effect and adverse birth outcomes in pregnant women with and without bacteriuria: a registry study
Source: Infection. 2025 Sep 10;53(6):2821–31. doi: 10.1007/s15010-025-02631-8 (PMC12675770; doi:10.1007/s15010-025-02631-8)
Supplement: Supplementary file 1 — Supplementary Material 1 [file 15010_2025_2631_MOESM1_ESM.docx]

**Supplementary Table S1:** Selected comorbidity and UTI ICD-10 codes

| **Comorbidity in analyses** | **ICD-10 codes** |
| --- | --- |
| Pyelonephritis | DN109C, DN129, DN118B, DN230, DO862B |
| Cystitis | DN30, DN300, DN308, DN309, DO231, DO862A |
| Urethritis | DN291, DN342, DN342G |
| Diabetes | DO240, DO240A, DO240B, DO240C, DO241, DO241A, DO241B, DO241C, DO243, DO243A, DO243B, DO243C, DO245, DO249, DO249, DO249B, DO249C, DE10, DE109, DE109A, DE11, DE119, DE119A, DO24. |
| Gestational diabetes mellitus (GDM) | DO244B, DO244C, DO244D, DO244E. |
| Chronic kidney disease (CKD) | DN18, DN181, DN182, DN183, DN184, DN185, DN189, DN03, DN039, DN259. |
| Hypertension | DI109, DI10, DI11, DI110, DI119, DI119A, DI15, DI158, DI159, DO139. |

**Supplementary Table S2:** Distribution of ICD-10 diagnoses in the study groups: ASB group and the UTI group.

| **Study groups ^1^** | UTI group  n = 533 | ASB group  n = 179 |
| --- | --- | --- |
| **ICD-10 diagnoses** | n (%) | n (%) |
| Cystitis **^2^** | 437 (69) | 29 (65) |
| Pyelonephritis **^3^** | 108 (17) | 10 (22) |
| Other UTIs **^4^** | 88 (14) | 6 (13) |
| Total diagnoses | 623 | 45 |
| No. of diagnosed pregnancies (% of study group diagnosed) | 533 (100) | 38 (21.2) |
| Diagnoses pr. pregnancy  (per diagnosed pregnancy) | 1.38 (1.38) | 1.18 (0.25) |

**^1^UTI group**: women with at least one significantly positive urine culture during pregnancy and an ICD-10 code for a UTI (n = 533). **ASB group**: women with at least one positive urine culture during pregnancy and an ICD-10 code for ASB (n = 179). **^2^**ICD-10: DO231, DN308, DN308c, DN309 (cystitis diagnoses). **^3^**ICD-10: DN109c, DN118b, DN129, DO230 (pyelonephritis diagnoses). **^4^**ICD-10: DN341, DO233, DO234 (urethritis, other UTI during pregnancy, UTI during pregnancy). **Note**: The analysis was performed using the open-source program R. **Note:** The bacteriuria group and the comparison group were not included, since they had no ICD-10 codes for ASB or UTI’s.

**Supplementary Table S3.1** Unadjusted logistic regression analysis on adverse birth outcomes (LBW, SGA and prematurity) among pregnancies with bacteriuria (n=2,786) including (top half) and excluding (bottom half) the comparison group.

|  | **LBW^1^** | |  | **SGA^1^** | | |  | | **Prematurity** | |  | | **Any outcome^1^** | |  | | |
| --- | --- | --- | --- | --- | --- | --- | --- | --- | --- | --- | --- | --- | --- | --- | --- | --- | --- |
| **Variable** | **cOR (95% CI)** | | **p** | **cOR (95% CI)** | | | **p** | | **cOR (95% CI)** | | **P** | | **cOR (95% CI)** | | **p** | | |
| Bacteriuria | 1.5 (1.3-1.7) | | <0.01 | 1.2 (1.1-1.4) | | | <0.01 | | 1.3 (1.1-1.5) | | <0.01 | | 1.3 (1.1-1.4) | | <0.01 | | |
| **Maternal data** |  |  | | |  |  | |  | |  | |  | | | |  |  |
| Age | 1.01 (0.99-1.02) | | 0.1 | 0.99 (0.99-1) | | | 0.06 | | 1 (0.99-1.01) | | 0.4 | | 1 (0.99-1.01) | | 0.1 | | |
| BMI | 1.003 (>1-1.007) | | 0.04 | 0.98 (0.97-0.98) | | | <0.01 | | >1 (~1-1.01) | | <0.01 | | 0.99 (0.98-0.99) | | <0.01 | | |
| Parity | 0.81 (0.8-0.9) | | <0.01 | 0.7 (0.66-0.72) | | | <0.01 | | 0.9 (0.8-0.92) | | <0.01 | | 0.75 (0.73-0.78) | | <0.01 | | |
| Smoking | 1.7 (1.5-1.9) | | <0.01 | 1.5 (1.4-1.6) | | | <0.01 | | 1.5 (1.3-1.6) | | <0.01 | | 1.5 (1.4-1.6) | | <0.01 | | |
| **Comorbidities** |  |  | | |  |  | |  | |  | |  | |  |  |  |  |
| Diabetes | 2.6 (1.9-3.5) | | <0.01 | 0.7 (0.5-1.1) | | | 0.06 | | 5.3 (4.3-6.6) | | <0.01 | | 2.3 (1.9-2.9) | | <0.01 | | |
| Hypertension | 2.3 (2.01-2.6) | | <0.01 | 1.5 (1.4-1.7) | | | <0.01 | | 1.8 (1.6-2) | | <0.01 | | 1.5 (1.4-1.6) | | <0.01 | | |
| CKD | 7 (4.13) | | <0.01 | 1.4 (0.7-2.9) | | | 0.4 | | 7.6 (4.5-13) | | <0.01 | | 3.5 (2.1-6) | | <0.01 | | |
| GDM | 1.1 (0.95-1.4) | | 0.2 | 0.9 (0.8-1.01) | | | 0.07 | | 1.4 (1.2-1.7) | | <0.01 | | 1.1 (0.97-1.2) | | 0.2 | | |
| **Variable** | **cOR (95% CI)** | | **p** | **cOR (95% CI)** | | | **p** | | **cOR (95% CI)** | | **P** | | **cOR (95% CI)** | | **p** | | |
| **Maternal data** |  | |  |  | | |  | |  | |  | |  | |  | | |
| Age | 1.05 (1.01-1.08) | | <0.01 | 1 (0.98-1.03) | | | 0.7 | | 1.01 (0.99-1.1) | | 0.1 | | 1.01 (0.99-1.03) | | 0.2 | |  |
| BMI | 1 (0.99-1.03) | | 0.3 | 0.98 (0.96-1.01) | | | 0.2 | | 1.01 (0.99-1.03) | | 0.06 | | 1 (0.99-1.02) | | 0.6 | |  |
| Parity | 1.3 (1.1-1.5) | | <0.01 | 0.76 (0.6-0.9) | | | <0.01 | | 1.1 (0.9-1.3) | | 0.3 | | 0.9 (0.8-1.03) | | 0.2 | |  |
| Smoking | 1.3 (0.8-2.1) | | 0.3 | 1.3 (0.9-1.9) | | | 0.2 | | 1.1 (0.7-1.8) | | 0.7 | | 1.3 (0.94-1.8) | | 0.1 | |  |
| **Comorbidities** |  |  | | |  |  | |  | |  | |  | |  |  |  |  |
| Diabetes | 3.2 (1.1-9.4) | | 0.04 | 0.3 (0.05-2.5) | | | 0.3 | | 13 (6-30) | | <0.01 | | 5.5 (2.5-12) | | <0.01 | | |
| Hypertension | 2.2 (1.2-3.7) | | <0.01 | 1.1 (0.6-1.8) | | | 0.8 | | 1.7 (0.99-3) | | >0.05 | | 1.2 (0.8-1.8) | | 0.5 | | |
| CKD | 8.4 (1.5-46) | | 0.02 | 1.6 (0.2-14) | | | 0.7 | | 13 (3-69) | | <0.01 | | 10 (1.8-55) | | <0.01 | | |
| GDM | 2.1 (1.2-3.7) | | <0.01 | 0.8 (0.4-1.4) | | | 0.4 | | 1.6 (0.9-2.8) | | 0.1 | | 0.99 (0.6-1.6) | | ~1 | | |

**^1^**LBW = Low birth weight, SGA = Small for gestational age. Any outcome = the occurrence of any of the three adverse birth outcomes (LBW, SGA and prematurity). **^2^**cOR: crude odds ratio. **^3^**CKD (chronic kidney disease). GDM (gestational diabetes mellitus). **Note**: The statistical analysis was performed using the open-source program R.

**Supplementary Table S3.2** Unadjusted logistic regression analysis on adverse birth outcomes (LBW, SGA and prematurity) among pregnancies with a urinary tract infection (UTI, n=533) including (top half) and excluding (bottom half) the comparison group.

|  | **LBW^1^** |  | **SGA^1^** |  | **Prematurity** |  | **Any outcome^1^** |  |
| --- | --- | --- | --- | --- | --- | --- | --- | --- |
| **Variable** | **cOR (95% CI)** | **p** | **cOR (95% CI)** | **p** | **cOR (95% CI)** | **P** | **cOR (95% CI)** | **p** |
| UTI | 2.7 (2-3.5) | <0.01 | 1.3 (0.97-1.6) | 0.08 | 2.5 (1.9-3.2) | <0.01 | 1.6 (1.3-2) | <0.01 |
| **Maternal data** |  |  |  |  |  |  |  |  |
| Age | 1 (0.99-1.01) | 0.2 | 0.99 (0.99-1) | 0.03 | 1 (0.99-1.01) | 0.6 | 0.99 (0.99-1) | 0.04 |
| BMI | 1 (1-1.01) | 0.08 | 0.98 (0.97-0.98) | <0.01 | 1 (1-1.01) | 0.02 | 0.99 (0.98-0.99) | <0.01 |
| Parity | 0.8 (0.75-0.84) | <0.01 | 0.7 (0.65-0.72) | <0.01 | 0.87 (0.8-0.9) | <0.01 | 0.75 (0.73-0.77) | <0.01 |
| Smoking | 1.7 (1.5-1.9) | <0.01 | 1.5 (1.4-1.6) | <0.01 | 1.5 (1.4-1.7) | <0.01 | 1.5 (1.4-1.65) | <0.01 |
| **Comorbidities** |  |  |  |  |  |  |  |  |
| Diabetes | 2.5 (1.9-3.4) | <0.01 | 0.74 (0.5-1.05) | 0.1 | 5 (4-6.1) | <0.01 | 2.2 (1.8-2.7) | <0.01 |
| Hypertension | 2.2 (2-.6) | <0.01 | 1.5 (1.4-1.7) | <0.01 | 1.8 (1.6-2) | <0.01 | 1.5 (1.4-1.6) | <0.01 |
| CKD | 7.1 (4-13) | <0.01 | 1.3 (0.6-2.9) | 0.5 | 7.3 (4.2-13) | <0.01 | 3.2 (1.9-5.6) | <0.01 |
| GDM | 1.06 (0.9-1.3) | 0.6 | 0.9 (0.8-1.01) | 0.06 | 1.4 (1.2-1.6) | <0.01 | 1.1 (0.96-1.2) | <0.01 |
| **Variable** | **cOR (95% CI)** | **p** | **cOR (95% CI)** | **p** | **cOR (95% CI)** | **P** | **cOR (95% CI)** | **p** |
| **Maternal data** |  |  |  |  |  |  |  |  |
| Age | 1.05 (1-1.1) | 0.045 | 0.98 (0.93-1.01) | 0.4 | 1.01 (0.98-1.01) | 0.2 | 0.99 (0.96-1.03) | 0.9 |
| BMI | 0.96 (0.9-1.01) | 0.2 | 0.99 (0.94-1.03) | 0.6 | 0.95 (0.9-1) | 0.07 | 0.98 (0.94-1.02) | 0.3 |
| Parity | 1.1 (0.8-1.5) | 0.53 | 0.9 (0.7-1.3) | 0.6 | 1.1 (0.8-1.4) | 0.6 | 1.05 (0.8-1.3) | 0.7 |
| Smoking | 1.5 (0.7-4.2) | 0.24 | 1.8 (0.9-3.5) | 0.1 | 2.5 (1.4-4.7) | <0.01 | 2.2 (1.3-3.8) | <0.01 |
| **Comorbidities** |  |  |  |  |  |  |  |  |
| Diabetes | 1.7 (0.4-7.9) | 0.53 | 0.6 (0.1-4.9) | 0.7 | 2.2 (0.6-8.2) | 0.2 | 1.2 (0.3-4.2) | 0.8 |
| Hypertension | 1.8 (0.7-4.8) | 0.3 | 1.1 (0.4-3.2) | 0.9 | 1.4 (0.5-3.7) | 0.5 | 1.1 (0.5-2.6) | 0.9 |
| CKD | NA^4^ | ~1 | NA | ~1 | NA | ~1 | NA | ~1 |
| GDM | 0.6 (0.1-2.6) | 0.5 | NA | ~1 | 0.5 (0.1-2) | 0.3 | 0.2 (0.06-1.03) | 0.06 |

**^1^**LBW = Low birth weight, SGA = Small for gestational age. Any outcome = the occurrence of any of the three adverse birth outcomes (LBW, SGA and prematurity). **^2^**cOR: crude odds ratio. **^3^**CKD (chronic kidney disease). GDM (gestational diabetes mellitus). **^4^**Highly skewed, inappropriate, and very insignificant results. **Note**: The statistical analysis was performed using the open-source program R.

**Supplementary Table S4:** Adjusted logistic regression analysis on adverse birth outcomes among pregnancies with a diagnosed urinary tract infection (UTI, 533 pregnancies with 623 ICD-10 UTI diagnoses) of Cystitis, Pyelonephritis, Other UTI) compared to women without bacteriuria, UTI and ASB (the comparison group; n = 70,755)

|  |  | **LBW ^1^** | | | | **SGA ^1^** | | | | **Prematurity** | | | |
| --- | --- | --- | --- | --- | --- | --- | --- | --- | --- | --- | --- | --- | --- |
| **ICD-10  codes** | **n** | **cOR ^2^**  **(95% CI)** | **P** | **aOR ^2^**  **(95% CI)** | **P** | **cOR (95% CI)** | **P** | **aOR (95% CI)** | **P** | **cOR (95% CI)** | **P** | **aOR (95% CI)** | **P** |
| **Cystitis ^4^** | 427 | 0.8 (0.5-1.5) | 0.5 | 2.5 (1.8-3.5) | <0.01 | 1.2 (0.7-2.1) | 0.5 | 1.3 (0.97-1.8) | 0.08 | 1.3 (0.8-2.2) | 0.4 | 2.5 (1.9-3.4) | <0.01 |
| **Pyelonephritis ^4^** | 108 | 1.1 (0.5-2.2) | 0.7 | 3.2 (1.7-5.8) | <0.01 | 0.7 (0.3-1.4) | 0.3 | 0.8 (0.4-1.7) | 0.6 | 0.9 (0.4-1.6) | 0.6 | 2.2 (1.2-4) | 0.01 |
| **Other UTI ^4^** | 88 | 1.2 (0.6-2.4) | 0.7 | 3.2 (1.6-6.2) | <0.01 | 1.1 (0.6-2.2) | 0.8 | 1.4 (0.97-1.8) | 0.3 | 0.8 (0.4-1.6) | 0.5 | 2 (1-4.1) | 0.05 |

**^1^**LBW = low birth weight. SGA = small for gestational age. **^2^**cOR = crude (unadjusted) odds ratio. aOR = adjusted odds ratio. Adjusted for: age, BMI, parity, diabetes, hypertension, chronic kidney disease (CKD), gestational diabetes mellitus (GDM) and smoking. **^4^**Selected ICD-10 diagnoses for each disease: Cystitis (DO231. DN308. DN308c. DN309). Pyelonephritis (DN109c. DN118b. DN129. DO230). Other UTI (DN341. DO233. DO234). **Note**: The statistical analysis was performed using the open-source program R.

**Supplementary table S5:** Number and prevalence of adverse birth outcomes in the study groups.

| **Group^1^** | **LBW^1^** n(%**^2^**) | **SGA^2^** n(%) | **Prematurity** n(%) | **Any outcome^2^** n(%) |
| --- | --- | --- | --- | --- |
| Bacteriuria | 159 (6) | 299 (11) | 192 (7) | 463 (17) |
| UTI | 52 (10) | 62 (12) | 65 (12) | 110 (21) |
| ASB | 8 (5) | 21 (12) | 9 (5) | 26 (15) |
| Comparison group | 2,831 (4) | 6,721 (10) | 3,820 (5) | 9,834 (14) |

^1^**Bacteriuria group**: women with at least one significantly positive urine culture during pregnancy and no ICD-10 code for ASB/UTI (n=2,786). **UTI group**: women with at least one significantly positive urine culture during pregnancy and an ICD-10 code for a UTI (n = 533). **ASB group**: women with at least one significantly positive urine culture during pregnancy and an ICD-10 code for ASB (n = 179). **Comparison group**: women without an ASB diagnosis, a UTI diagnosis or bacteriuria (n = 70,755). ^2^LBW = low birth weight, SGA = small for gestational age, Any outcome = the occurrence of any of the three adverse birth outcomes (LBW, SGA and prematurity). **^2^**Percentage of pregnancies in the study group with the given outcome.

**Supplementary table S6:** **Adjusted logistic regression analysis on adverse birth outcomes (LBW, SGA, and prematurity) among pregnancies with asymptomatic bacteriuria (ASB; n = 179) compared to women without a significantly positive urine culture (the comparison group; n=70,755).**

|  | **Pregnancies with an ICD-10 code for ASB**  **(n=179)** | | | | | | | | |
| --- | --- | --- | --- | --- | --- | --- | --- | --- | --- |
|  | **LBW^1^** | | **SGA^1^** | | | **Prematurity** | | **Any outcome^1^** | |
| **Variable** | aOR^2^  (95% CI) | *p* | aOR  (95% CI) | *p* | aOR  (95% CI) | | *p* | aOR  (95% CI) | *p* |
| Group | 1.03  (0.45-2.4) | 0.93 | 1.33  (0.8-2.2) | 0.27 | 0.73  (0.3-1.7) | | 0.46 | 1.06  (0.7-1.55) | 0.98 |
| **Maternal data** |  |  |  |  |  | |  |  |  |
| Age | 1.02 (1.01-1.1) | <0.01 | 1.01  (1.01-1.02) | <0.01 | 1.01  (1.01-1.02) | | 0.01 | 1.01  (1-1.02) | <0.01 |
| BMI | 1  (1-1.01) | <0.01 | 0.98  (0.97-0.98) | <0.01 | 1 (1-1.01) | | 0.13 | 0.99  (0.98-0.99) | <0.01 |
| Parity | 0.76  (0.7-0.8) | <0.01 | 0.7  (0.6-0.71) | <0.01 | 0.84  (0.8-0.9) | | <0.01 | 0.73  (0.7-0.75) | <0.01 |
| Smoking | 1.8  (1.6-2) | <0.01 | 1.5 (1.4-1.7) | <0.01 | 1.5 (1.3-1.7) | | <0.01 | 1.5  (1.4-1.7) | <0.01 |
| **Comorbidities** |  |  |  |  |  | |  |  |  |
| Diabetes | 2.2  (1.6-3) | <0.01 | 0.7  (0.5-1.02) | 0.06 | 4.6 (3.7-5.7) | | <0.01 | 2.2  (1.8-2.7) | <0.01 |
| Hypertension | 2.1  (1.9-2.5) | <0.01 | 1.6  (1.4-1.8) | <0.01 | 1.6 (1.4-1.7) | | <0.01 | 1.5  (1.3-1.6) | <0.01 |
| CKD | 5  (2.7-9.1) | <0.01 | 1.1  (0.5-2.4) | 0.8 | 7 (4-11.8) | | <0.01 | 2.7  (1.5-4.6) | <0.01 |
| GDM | 0.99  (0.8-1.2) | 0.98 | 1.04  (0.9-1.2) | 0.6 | 1.2 (1.1-1.4) | | 0.01 | 1.1  (0.98-1.2) | 0.08 |

**^1^**LBW = Low birth weight, SGA = Small for gestational age, Any outcome = the occurrence of any of the three adverse birth outcomes (LBW, SGA and prematurity).**^2^**aOR (adjusted odds ratio) adjusted for**:** age, BMI, parity, diabetes mellitus, hypertension, chronic kidney disease (CKD), gestational diabetes mellitus (GDM), and smoking. **Note**: The statistical analysis was performed using the open-source program R.

**Supplementary table S7: Adjusted logistic regression showing the effect of treatment of ASB on adverse birth outcomes (LBW, SGA, and prematurity) among pregnancies with ASB (n=179)**

|  |  | **LBW ^1^** | | **SGA ^1^** | | **Prematurity** | | **Any adverse birth outcome ^1^** | |
| --- | --- | --- | --- | --- | --- | --- | --- | --- | --- |
| **Group** | **n** | aOR ^2^ (95% CI) | *P* | aOR (95% CI) | *P* | aOR (95% CI) | *P* | aOR (95% CI) | *P* |
| ASB ^4^ | 179 | NA (NA) ^3^ | ~1 | 0.7 (0.1-6.2) | 0.7 | NA (NA) | ~1 | 0.9 (0.1-7.7) | 0.9 |

**^1^**LBW = Low birth weight, SGA = Small for gestational age, Any outcome = the occurrence of any of the three adverse birth outcomes (LBW, SGA and prematurity). **^2^**aOR (adjusted odds ratio) adjusted for**:** age, BMI, parity, diabetes mellitus, hypertension, chronic kidney disease (CKD), gestational diabetes mellitus (GDM), and smoking. **^3^**Highly skewed, invalid, and very insignificant results. **^4^**ASB (asymptomatic bacteriuria, significantly positive urine culture and an ICD-10 coded for ASB). **Note**: The statistical analysis was performed using the open-source program R.
